# Characterizing Properdin-Inhibited C3 Nephritic Factors in Patients with Complement-Mediated Kidney Diseases

**DOI:** 10.3390/ijms27156790

**Published:** 2026-07-29

**Authors:** Kes H. Stevens, Rianne J. F. Maas, Elena B. Volokhina, Erik J. M. Toonen, Nicole C. A. J. van de Kar, Lambertus P. W. J. van den Heuvel, Marloes A. H. M. Michels

**Affiliations:** 1 Department of Pediatric Nephrology, Amalia Children’s Hospital, Radboud University Medical Center, 6525 Nijmegen, The Netherlands; 2 R&D Department, Hycult Biotech, 5405 Uden, The Netherlands; 3 Department of Human Genetics, Radboud University Medical Center, 6525 Nijmegen, The Netherlands; 4 Department of Pediatrics/Pediatric Nephrology, University Hospitals Leuven, 3000 Leuven, Belgium; 5 Department of Development and Regeneration, Catholic University Leuven, 3000 Leuven, Belgium

**Keywords:** C3 glomerulopathy, C3 nephritic factors, complement system, properdin, Salp20, alternative pathway

## Abstract

C3 glomerulopathy (C3G) and immune complex-mediated membranoproliferative glomerulonephritis (IC-MPGN) are severe complement-mediated kidney diseases. In a substantial proportion of these patients, C3 nephritic factors (C3NeFs) are detected; these autoantibodies stabilize the complement alternative pathway (AP) C3 convertase. Previous studies have investigated and distinguished properdin-dependent and properdin-independent C3NeFs. In this study, we investigated a distinct subset of C3NeFs that are exclusively active in the absence of properdin: properdin-inhibited C3NeFs. A two-step hemolytic AP convertase activity assay was employed to examine convertase activity in patient serum in the presence versus absence of properdin using the properdin inhibitor Salp20. In 7/16 patients and 1/23 healthy controls, who showed no convertase stabilization in full serum, convertase stabilization was observed upon properdin inhibition, indicating properdin-inhibited C3NeF activity. Consistent findings were obtained when purified patient Igs were added to properdin-depleted serum. Moreover, in an ELISA-based C3bBb binding assay, 6/7 patients with properdin-inhibited C3NeFs showed increased convertase binding, which decreased upon addition of properdin. Complement levels in patients with properdin-inhibited C3NeFs were not significantly different from those in the C3NeF-negative group, and properdin levels were generally within the refence range. In conclusion, our results support the existence of properdin-inhibited C3NeFs, thereby further expanding the functional heterogeneity of these autoantibodies. Although their functional relevance under physiological conditions remains unclear, these findings may have important implications for complement-targeted therapies, particularly strategies aimed at properdin inhibition, as the presence of these C3NeFs may predispose to unintended effects.

## 1. Introduction

The complement system is an important part of the innate immune system and plays a pivotal role in the protection of the human body from pathogens and dangerous host cells. Activation can occur via three pathways: the classical pathway, the lectin pathway, and the alternative pathway (AP). Activation of these pathways depends on the initial trigger, but all pathways lead to the formation of a C3 convertase, which will cleave complement C3 and induce further activation of the complement cascade. As a result, anaphylatoxins, opsonins, and the terminal complement complex (TCC) will be formed, supporting inflammation, phagocytosis, and cell damage [1,2].

In contrast to the lectin pathway and classical pathway, which are specifically activated upon pattern recognition, the AP is continuously active at a low level, making the AP an important surveillance mechanism. In a small percentage of the C3 molecules, the internal thioester bond is spontaneously hydrolyzed, generating C3(H_2_O). The hydrolyzed C3 can be bound by Factor B (FB). Upon FB cleavage by Factor D (FD), the fluid-phase C3 convertase, C3(H_2_O)Bb, is formed. This complex cleaves small amounts of C3 into C3a and C3b. The C3b can bind to (bacterial) cell surfaces, leading to opsonization. Moreover, membrane-bound C3b can be used to form new C3 convertases with FB and FD, i.e., C3bBb. These convertases can be stabilized by the glycoprotein properdin, the positive regulator of the AP [3,4]. By binding to C3 convertases, properdin increases the half-life of these complexes five- to ten-fold [5]. The formed C3bBbP complexes generate additional C3b, thereby promoting further convertase formation and amplification of the cascade. Through this potent amplification mechanism, the C3b density on the cell surface increases, resulting in C5 convertase activity [6,7]. The C5 convertases cleave C5 into C5a and C5b, initiating the terminal pathway. C5b will assemble with one unit of complement components C6 to C8 and multiple units of C9 to form the lytic TCC, the end product of complement activity [1,2].

The activity of the AP is strictly regulated by various negative regulators. Membrane-bound and circulating inhibitory proteins, such as Factor H, Factor I, complement receptor 1 (CD35), membrane cofactor protein (CD46), and decay-accelerating factor (CD55), break down the convertases or prevent the formation of new convertases. Thereby, excessive AP activation and subsequent damage to healthy host cells are prevented [2,8]. However, when pathogenic autoantibodies or mutations in AP (regulatory) proteins disrupt the balance between convertase activation and inhibition, the AP may become hyperactive. As a result, healthy tissues, especially in the kidneys, are injured [9,10,11], which may lead to severe complement-mediated kidney diseases such as C3 glomerulopathy (C3G) [11,12,13].

C3G patients can present with various symptoms, such as hematuria, proteinuria, hypertension, and low serum C3. The diagnosis is based on a kidney biopsy showing C3 dominance with immune fluorescence, reflecting the complement AP overactivation. C3G encompasses two subtypes that have different electron microscopy appearances: dense deposit disease (DDD) and C3 glomerulonephritis (C3GN) [12,14,15]. DDD is characterized by very dense band-like intramembranous deposits, whereas the deposits found in C3GN are less dense and can show a more variable pattern [12,14]. The general prognosis for C3G is poor, with about 50% of all patients developing end-stage kidney disease within ten years after diagnosis and a risk of approximately 65% of recurrence in kidney transplants [16,17,18,19,20,21,22,23,24]. Standard treatment includes general immunosuppression and antihypertensive drugs [12,25]: however, these nonspecific approaches have demonstrated limited efficacy [12,26,27].

Given that the disease is driven by AP dysregulation and complement overactivation, targeting the complement system has emerged as a rational therapeutic strategy. In 2025, pegcetacoplan (C3 inhibitor) and iptacopan (FB inhibitor) were approved by the US Food and Drug Administration for clinical use in C3G patients [28], and several other complement inhibitors are currently in clinical trials [29]. For optimizing complement-inhibiting therapy and guiding individualized treatment strategies, accurate assessment and characterization of complement activation in these patients is essential.

In up to 20% of the C3G patients, rare variants in complement genes have been found [13,18,21,30]. In addition, acquired autoantibodies targeting complement factors have been shown to contribute to disease. The most prominent autoantibodies in C3G are C3 nephritic factors (C3NeFs), which are detected in ∼40–50% of C3GN patients and in ∼60–80% of DDD patients [31]. C3NeFs prolong the activity of the AP C3 convertase by inhibiting the natural intrinsic decay and accelerated convertase decay mediated by complement regulators [32,33,34,35]. Thereby, these autoantibodies contribute to uncontrolled AP activation and subsequent injury to healthy kidney tissues. C3NeFs are not only detected in C3G, but also in immune complex-mediated membranoproliferative glomerulonephritis (IC-MPGN), which is diagnostically distinguished from C3G by the presence of immunoglobulin (Ig) deposits in the glomerulus [18,36,37].

**Figure 1:**
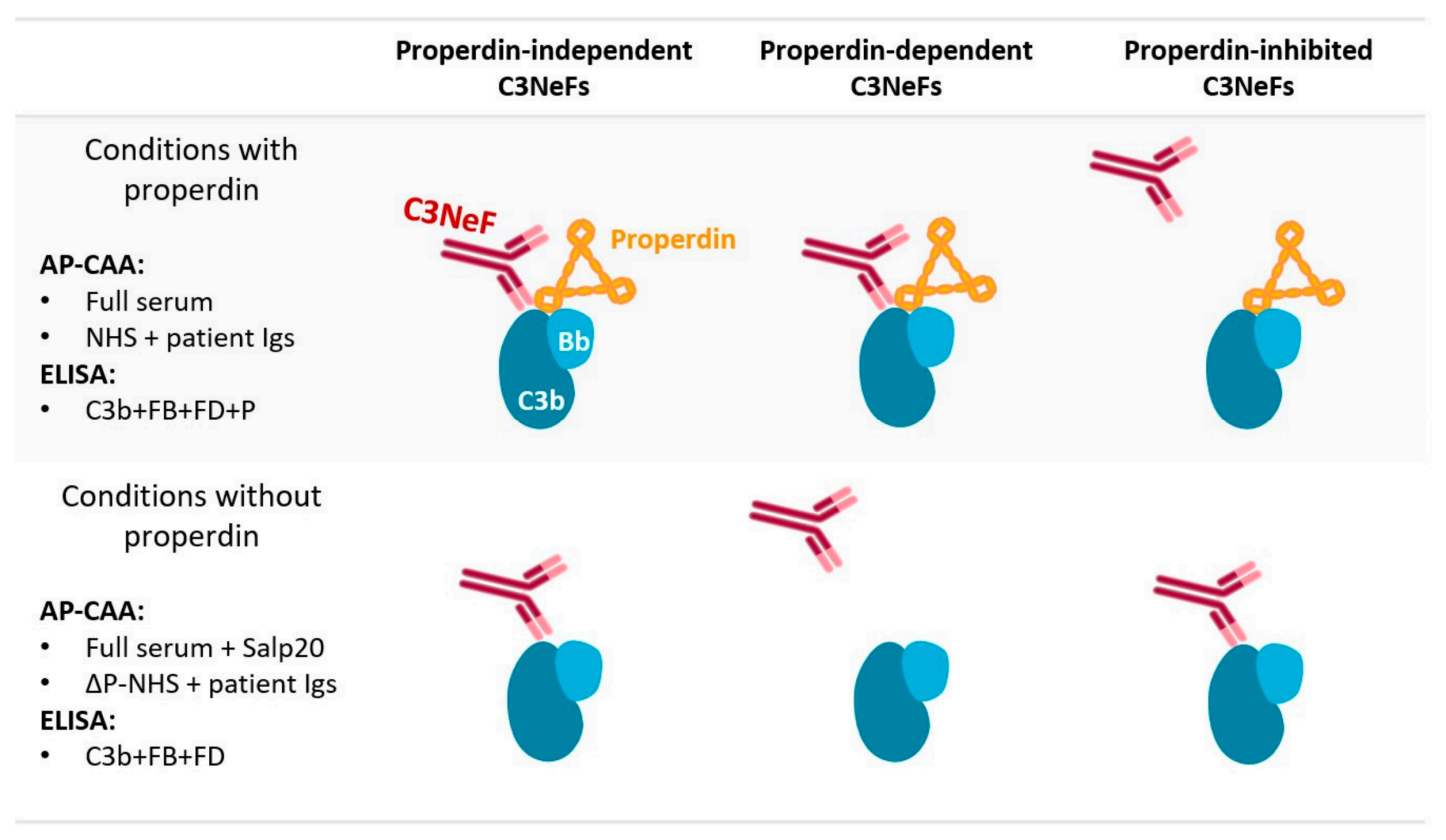
Schematic overview of the different types of C3 nephritic factors (C3NeFs). In this study, the alternative pathway (AP)-convertase activity assay (CAA) and anti-C3bBb(P) detection ELISA were employed to study the interactions between the different C3NeF types and the AP convertase with or without properdin, by modulating experimental conditions. FB, Factor B; FD, Factor D; Igs, immunoglobulins; ΔP-NHS, properdin-depleted NHS.

C3NeFs are heterogeneous in their mode of action. For example, two classes of C3NeFs are distinguished: properdin-dependent and properdin-independent C3NeFs. To identify and distinguish between these two C3NeF-subtypes, assays test for C3NeF activity (i.e., convertase stabilization) in a condition with and without properdin (Figure 1). Properdin-dependent C3NeFs are only active in the presence of properdin (stabilizing C3bBbP(C3b) but not C3bBb), whereas properdin-independent C3NeFs are active both in the presence and absence of properdin (stabilizing both C3bBbP(C3b) and C3bBb) [17,34,38,39,40,41]. The properdin-containing condition, which most closely reflects physiological circumstances, identifies the highest number of patients with C3NeFs [34,40]. Notably, these approaches have also enabled the detection of C3NeFs that were active exclusively in the absence of properdin and inactive when properdin was present [17,42]. However, the characterization of these particular C3NeFs is limited.

In the context of emerging complement-targeted therapies, particularly properdin-inhibiting strategies [4,43,44,45], precise characterization of patients and their specific C3NeF profiles is essential. Parallel to our previous approach to distinguish properdin-dependent from properdin-independent C3NeFs [41], in this study we investigated the subclass of C3NeFs that are exclusively active in the absence of properdin. We will refer to them as properdin-inhibited C3NeFs, as it seems that presence of properdin inhibits the convertase-stabilizing actions of these C3NeFs (Figure 1). We selected C3G/IC-MPGN patients who were previously classified negative for properdin-dependent and properdin-independent C3NeF activity and assessed the presence of properdin-inhibited C3NeF activity using different assay conditions lacking properdin. Furthermore, we explored the functional relevance of these properdin-inhibited C3NeFs.

## 2. Results

### 2.1. Patient Characteristics

For this study, we selected patients from a retrospective C3G/IC-MPGN cohort who had previously been found negative for prolonged convertase activity, i.e., C3NeF activity, in test conditions of whole serum [46,47,48]. For sixteen patients, material was available for this study: twelve C3G patients (four C3GN, six DDD, and two with unknown subtype) and four IC-MPGN patients (Table 1). The mean age of the patients at time of study was 29 years (range: 9–66 years; seven children, nine adults).

### 2.2. Determining Normal Convertase Activity in the Absence of Properdin Using Serum and Immunoglobulins of Healthy Controls

Before screening patients for C3NeF activity in the absence of properdin, serum and Igs from healthy controls were tested in the AP-convertase activity assay (CAA) to establish cut-off values for normal convertase activity in conditions without properdin. The convertase activity profile in the absence of properdin is characterized by maximal convertase activity around 30 min, followed by convertase breakdown and hemolysis returning to background levels at 50 and 60 min. Therefore, our cut-off values were based on differences in hemolysis of the test sample vs the internal control at time points t50 and t60 (see Methods Section 4.3).

**Figure 2:**
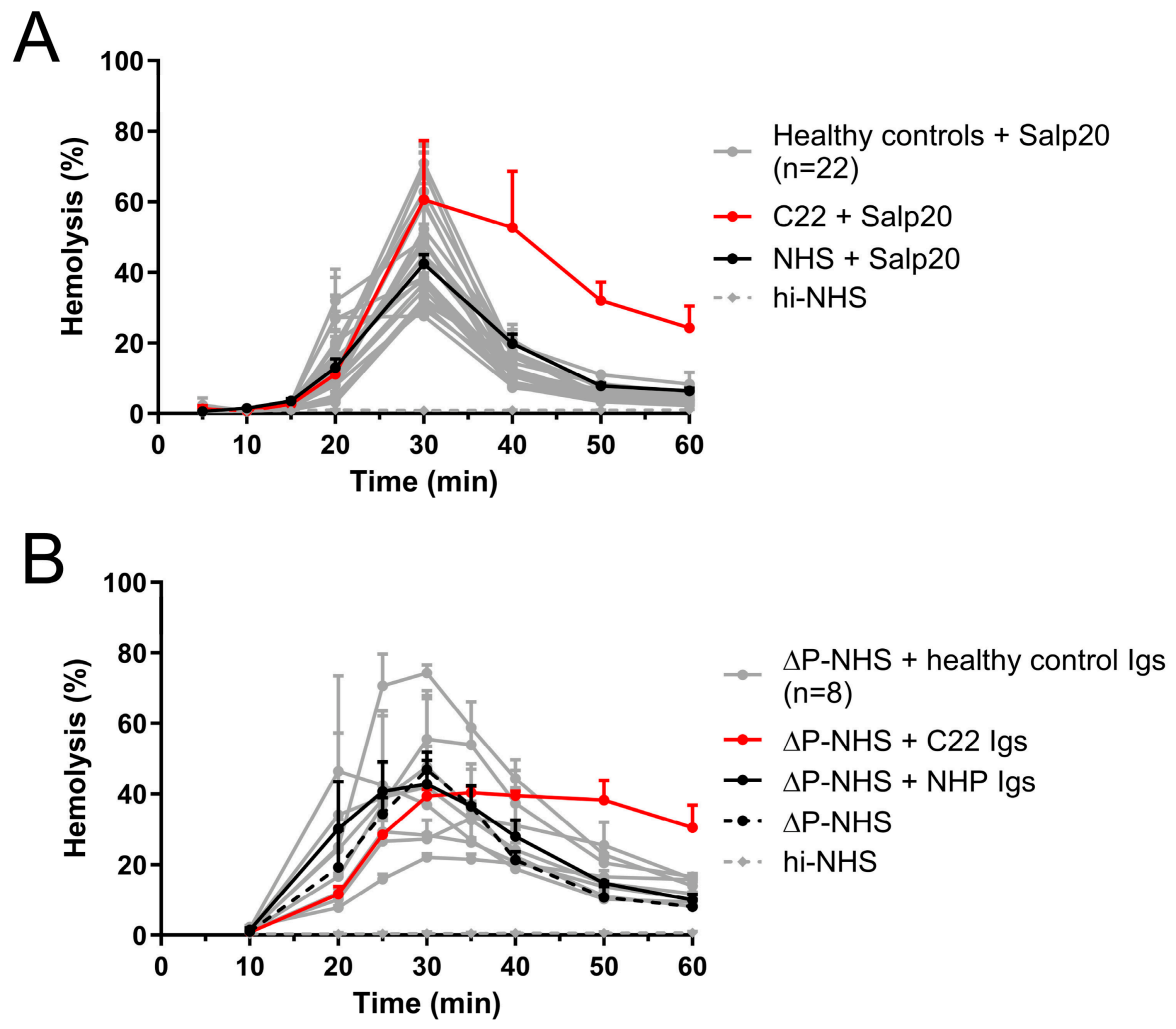
Convertase activity profiles of healthy controls in the absence of properdin. The AP-CAA was performed with healthy control serum supplemented with the properdin-inhibitor Salp20 (**A**) or with Igs fractions that were added to ΔP-NHS (**B**). As an internal control, Igs originating from normal human plasma (NHP) were used. The healthy controls used for determination of cut-off values are shown in grey. Healthy control 22 (C22) was excluded for the determination of cut-off values (shown in red). Data are presented as mean + range of two independent experiments. hi-NHS, heat-inactivated NHS.

In the absence of properdin, i.e., by adding 6.25 µg/mL of the properdin inhibitor Salp20 [41], twenty-two healthy controls showed a convertase activity profile that was similar to NHS with Salp20 (Figure 2A; mean relative deviation to NHS + Salp20 at t50 + t60 = 0.66, SD = 0.18; cut-off = 1.20)). Surprisingly, one healthy control (C22) displayed prolonged convertase activity when properdin was blocked. The serum of C22 without Salp20 inhibition, however, showed normal convertase activity (Figure S1A). C22 was identified as an outlier (see Methods Section 4.3) in the condition of serum with Salp20 and was therefore excluded from all analyses involving cut-off determination. The convertase activity profiles induced by the nine available healthy control Ig fractions in properdin-depleted NHS (ΔP-NHS; mean relative deviation to ΔP-NHS + NHP-Igs = 1.16, SD = 0.26, cut-off = 1.95) supported the findings of the serum conditions with Salp20 (Figure 2B). Again, C22 Igs showed increased convertase stability in ΔP-NHS, whereas this was not seen when the Igs were added to NHS (Figure S1B).

### 2.3. Screening Patient Samples for Properdin-Inhibited C3NeF Activity in Serum Using Salp20

**Figure 3:**
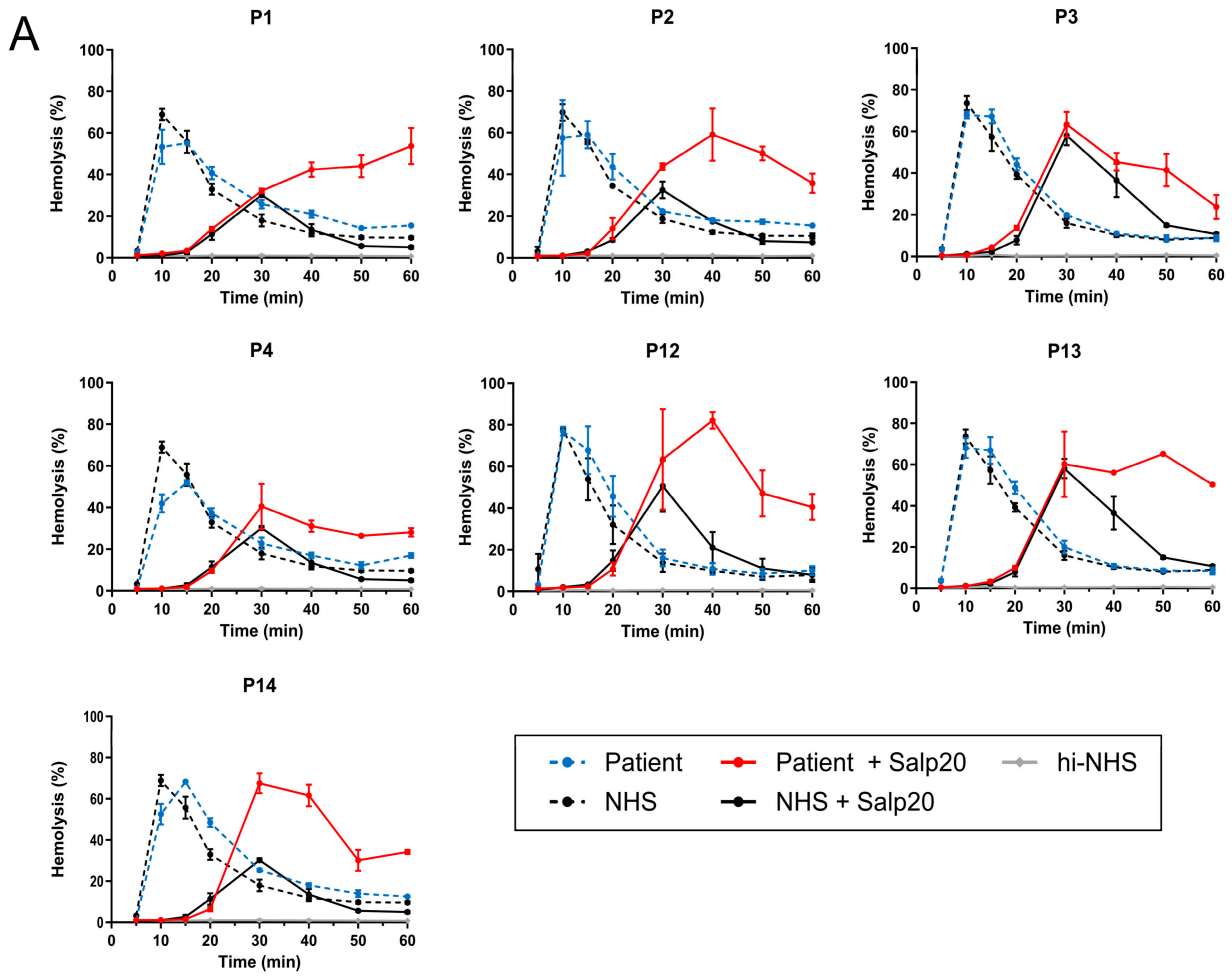

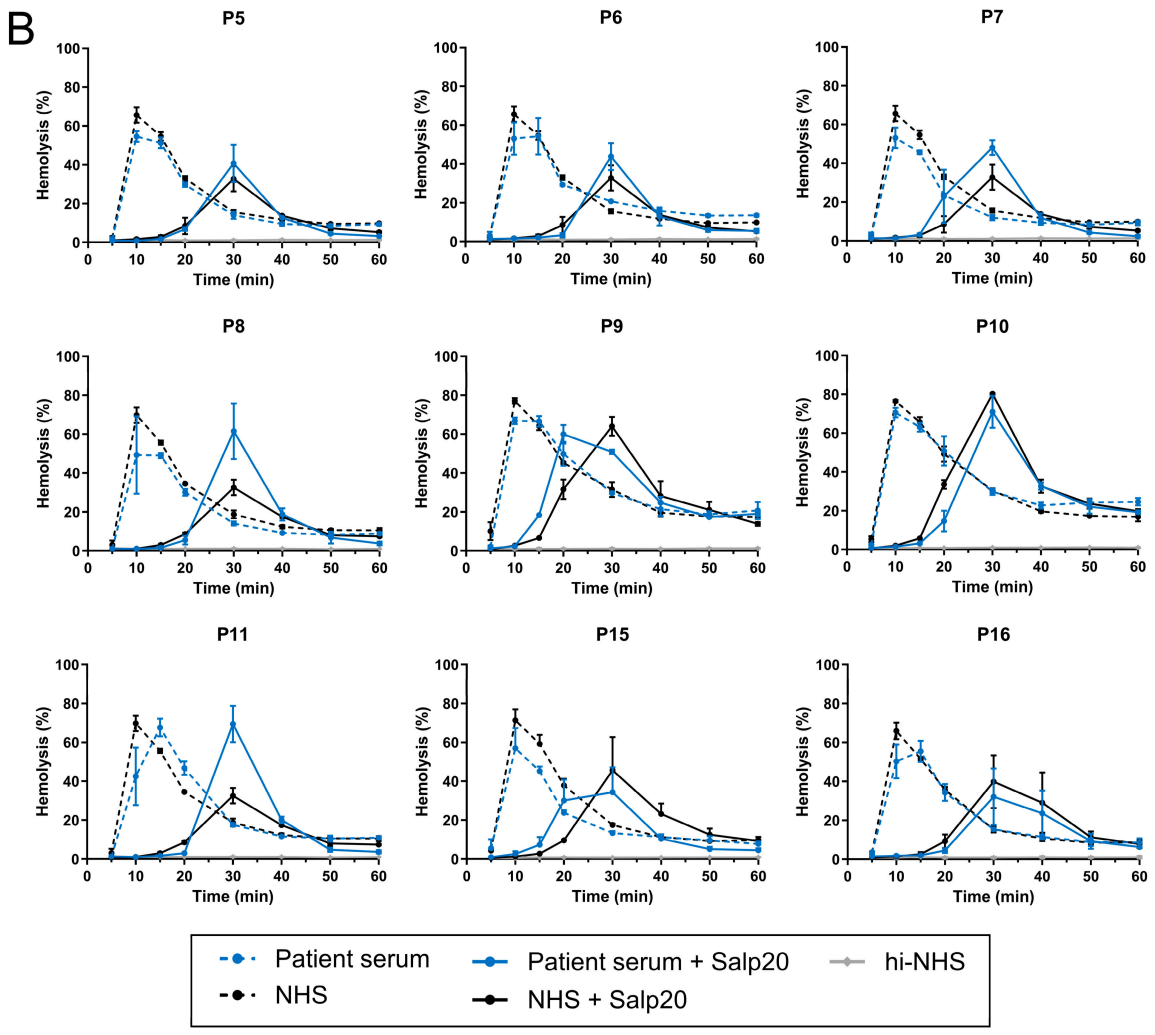
Screening patient samples for properdin-inhibited C3NeF activity using Salp20. AP-CAA was performed with patient serum (P1, P3–8, P10, P11, and P13–15) or patient ethylenediamine-tetraacetic acid (EDTA)-plasma (P2, P9, P12, and P16), supplemented or not with the properdin inhibitor Salp20. (**A**) Patients positive for the properdin-inhibited C3NeF. (**B**) Patients negative for the properdin-inhibited C3NeF. Blue and red lines indicate convertase activity profiles of patients within and outside the mean relative deviation to the internal control (NHS or NHS + Salp20) + 3SD of healthy controls, respectively. Data are presented as mean ± range from two independent experiments.

Next, patient sera or plasma were screened for prolonged convertase activity in the AP-CAA in the presence and absence of properdin using properdin inhibitor Salp20. In the absence of properdin, convertase activity was significantly different in the patients compared to the controls (Figure S2A), while this was not the case in full serum conditions with properdin (Figure S2B). Prolonged convertase activity, according to our cut-off values, was observed in seven of the sixteen patients (P1–4, P12–14; Figure 3A; relative deviation to NHS + Salp20 > 1.20). In complete serum conditions, none of the patients showed convertase stabilization, which is in line with our inclusion criteria. This suggests the presence of properdin-inhibited C3NeFs in these seven patient samples, as the convertase-stabilizing activity could only be detected upon properdin inhibition. The other nine patients showed normal convertase activity profiles both with and without properdin inhibition (P5–P11, P15, P16, Figure 3B), indicating that these patients have no C3NeF activity at all.

### 2.4. Screening Patient Immunoglobulins for Properdin-Inhibited C3NeFs Using ΔP-NHS

**Figure 4:**
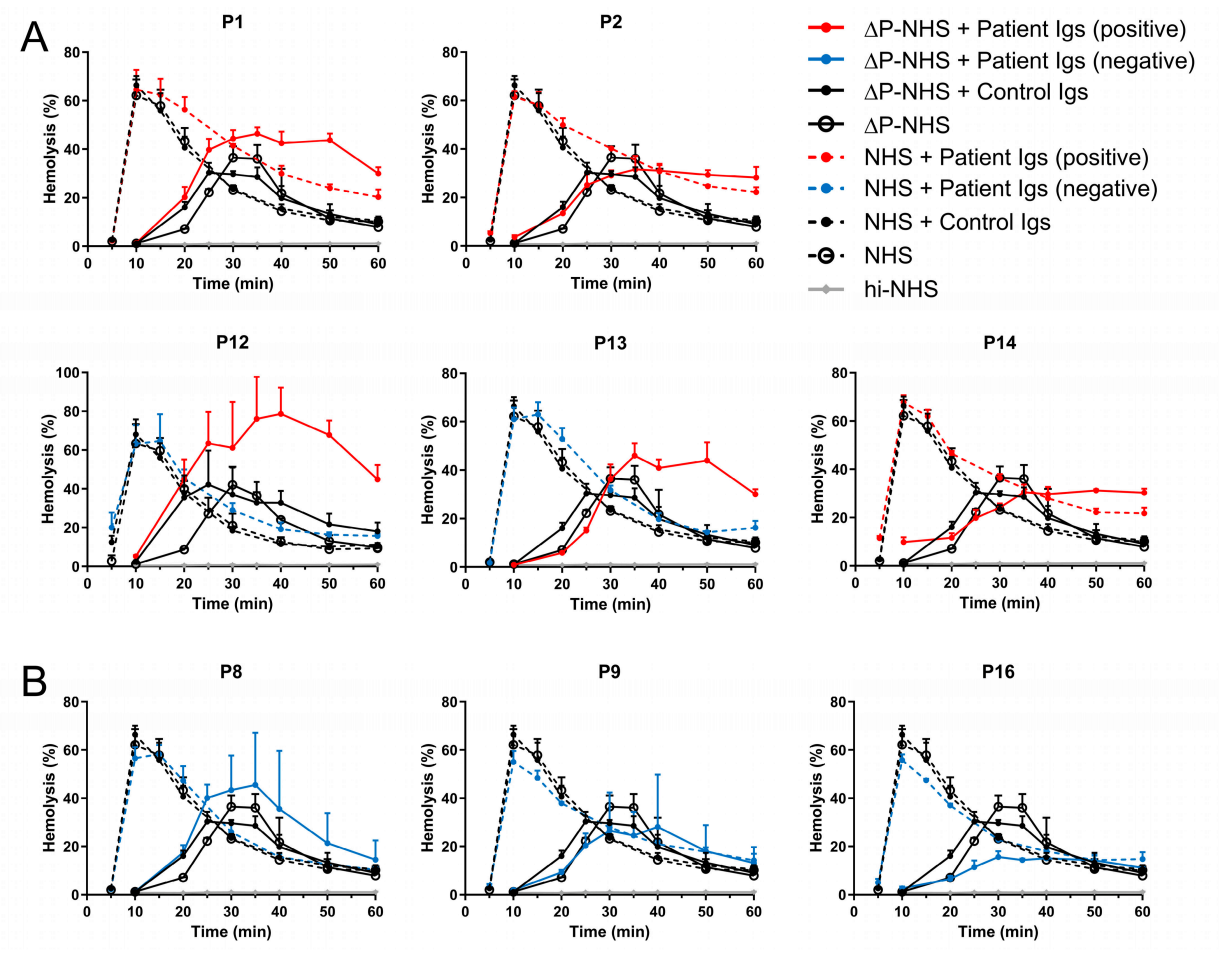
Screening patient Ig fractions for properdin-inhibited C3NeFs using ΔP-NHS. AP-CAA was performed with NHS or ΔP-NHS supplemented with Igs originating from patients positive for properdin-inhibited C3NeFs (**A**) or negative for C3NeFs (**B**). As an internal control, Igs originating from pooled NHP or NHS (control Igs) were used, depending on the patient material used for Ig purification. Blue and red lines indicate convertase activity profiles with patient Igs within and outside the mean relative deviation to the internal control (NHS + Control Igs or ΔP-NHS + Control Igs) + 3SD of healthy controls, respectively. Data are presented as mean + SD from three independent experiments.

To confirm the autoantibody nature of the factors that induced the convertase stabilization in the patient samples in the absence of properdin, we added the purified Ig fractions from these samples to ΔP-NHS. Sufficient material was available for five of the seven (71%) patients, i.e., P1, P2, and P12–14. Indeed, the Ig fractions from these patients with prolonged convertase activity in properdin-blocked serum also induced convertase stabilization in ΔP-NHS (Figure 4A; relative deviation to ΔP-NHS + NHP-Igs > 1.95). This further supported that a properdin-inhibited C3NeF induced the prolonged convertase activity in the absence of properdin.

Unexpectedly, the Ig fractions of P1, P2, and P14, did also induce slightly prolonged convertase activity when added to NHS (Figure 4A; relative deviation to NHS + NHP-Igs > 1.36), whereas all patient sera showed normal convertase activity profiles (Figure 3A). However, the degree of convertase stabilization induced by P1, P2, and P14 Igs seems to be less pronounced in the presence of properdin (when added to NHS) compared to the convertase stabilization in the absence of properdin (when added to ΔP-NHS).

From three of the nine (33%) patients who were C3NeF-negative in both full serum and properdin-blocked serum conditions, i.e., P8, P9, and P16, Ig fractions were available. When added to NHS and ΔP-NHS, these Igs showed normal convertase activity profiles (Figure 4B), which is in line with our previous findings of patient serum with and without properdin inhibition by Salp20.

### 2.5. Identifying and Characterizing Properdin-Inhibited C3NeFs by IgG Binding to C3bBb

**Figure 5:**
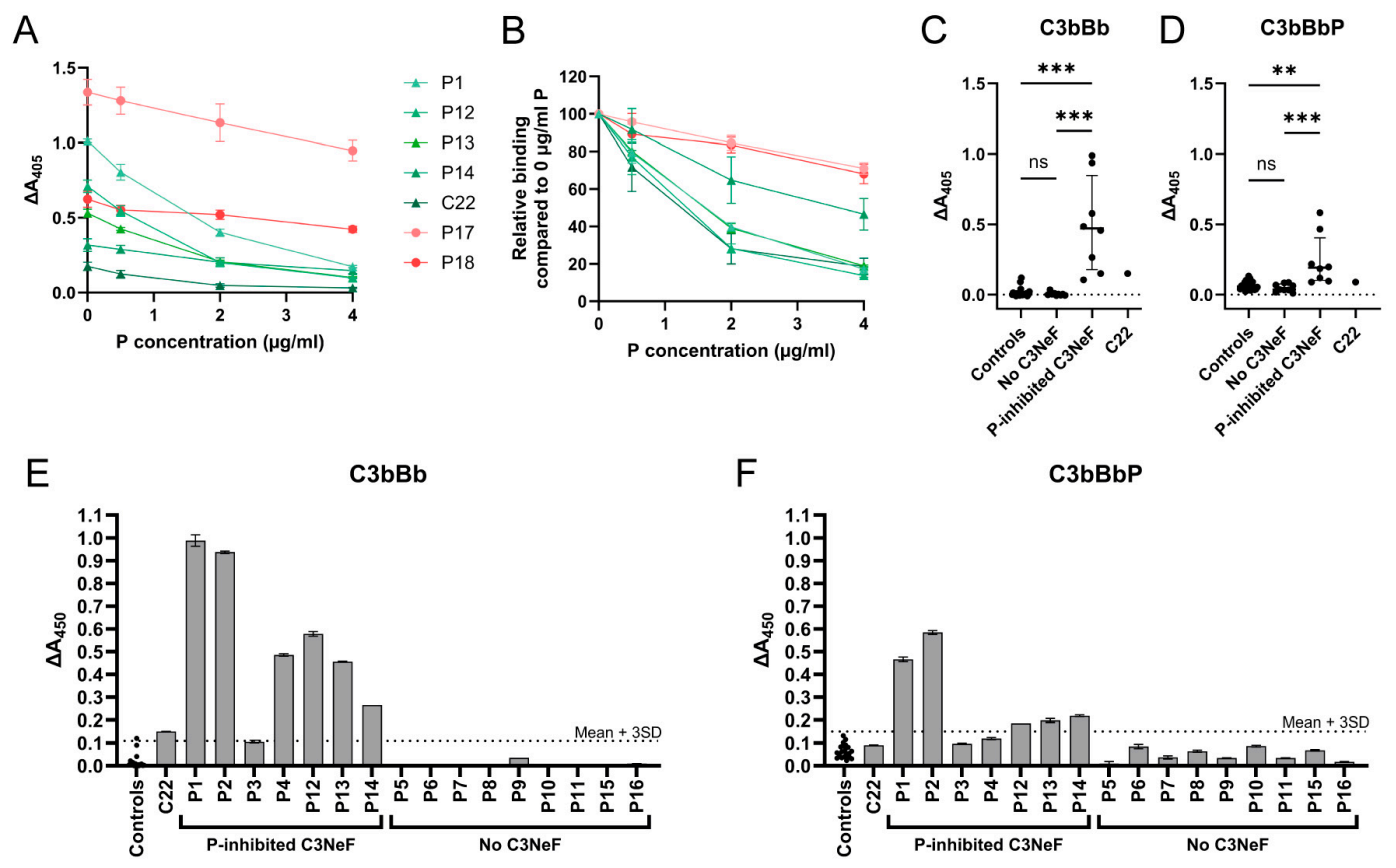
Screening for IgG binding to the C3 convertase in the presence and absence of properdin (P). Samples were categorized based on their outcome in the AP-CAA. (**A**) Samples positive for P-independent C3NeF (red circles) and positive for P-inhibited C3NeF (green triangles) were tested without and with different P concentrations. (**B**) Delta absorbance values (ΔA_405_ values) were transformed to relative binding in percentage compared to the condition without P. (**A**,**B**) Data are presented as mean ± SD of three independent experiments. (**C**–**F**) C22 was visualized separately as this is a healthy control positive for the properdin-inhibited C3NeFs according to the AP-CAA. ΔA_405_ values of the test samples without (C3bBb; (**C**,**E**)) and with (C3bBbP; (**D**,**F**)) P. (**C**,**D**) Data are presented as median ± interquartile range. Not significant (ns), ** *p* < 0.01, *** *p* < 0.001, using Kruskal–Wallis test with Dunn’s multiple comparisons tests. (**E**,**F**) Individual visualization of the patients and C22 of panel (**C**,**D**). (**E**,**F**) For the patients and C22, data are presented as mean ± range from two replicates in a single experiment. For the healthy controls, each dot represents the mean from two replicates in a single experiment of one individual healthy control. The dotted line represent the mean + 3SD of 22 healthy controls.

To confirm the presence of properdin-inhibited C3NeFs with an alternative approach, an ELISA-based method was used to assess direct IgG binding of properdin-inhibited C3NeFs to C3bBb. Five samples (C22, P1, P12–14) positive for properdin-inhibited C3NeFs and two properdin-independent C3NeF samples (P17 and P18, as determined in ref. [41]) were analyzed for IgG binding to the convertase, both in the absence (C3bBb) and presence (C3bBbP) of properdin. Upon addition of properdin, binding to the convertase decreased in a concentration-dependent manner across all samples (Figure 5A,B); however, the magnitude of this effect differed significantly between patients (*p* < 0.0001), indicating heterogeneous sensitivity for properdin. Nonlinear regression analysis revealed a broad range of half maximal inhibitory concentration (IC_50_) values for properdin on properdin-inhibited C3NeF activity (0.98–10.17 µg/mL; Table 2). Samples classified as properdin-inhibited C3NeFs exhibited low IC_50_ values (0.98–3.69 µg/mL), reflecting high sensitivity to properdin, whereas properdin-independent C3NeFs showed markedly higher IC_50_ values (8.63–10.17 µg/mL), indicating reduced sensitivity. These findings demonstrate that properdin-inhibited C3NeFs are more strongly modulated at lower properdin concentrations, while properdin-independent C3NeFs require higher properdin levels to achieve comparable effects.

Next, all patient samples from the study cohort, along with the healthy control samples, were analyzed using ELISA without and with 2 µg/mL properdin. The seven patients positive for properdin-inhibited C3NeFs according to the AP-CAA results showed significantly higher C3bBb binding compared to both the healthy controls and the patient group negative for C3NeFs (Figure 5C). Although decreased convertase binding was observed when properdin was added to properdin-inhibited C3NeFs (Figure 5A,B), these samples still exhibited significantly higher C3bBbP binding compared to both the healthy controls and C3NeF-negative group (Figure 5D).

To investigate the diagnostic value of the C3bBb and C3bBbP binding ELISA for the group of properdin-inhibited C3NeFs, we studied the convertase binding of the patients individually compared to healthy controls. Six out of the seven patients (86%; P1, P2, P4, P12, P13, and P14) with properdin-inhibited C3NeFs showed increased C3bBb binding (Figure 5E; ΔA_405_ > 0.11). Although the signal was higher in the C3bBb condition, 5/7 (71%; P1, P2, P12–14) properdin-inhibited samples were also positive for C3bBbP binding (Figure 5F; ΔA_405_ > 0.15). All samples that were negative in the absence of properdin (Figure 5E) remained negative when properdin was present (Figure 5F). These results indicate that properdin-inhibited C3NeF-positive samples are not detected by the standard AP-CAA in full serum conditions, while the majority were detected with the ELISA-based method both in the presence and absence of properdin.

### 2.6. Examining the Effect of the Properdin-Inhibited C3NeFs on the Complement Profile

**Figure 6:**
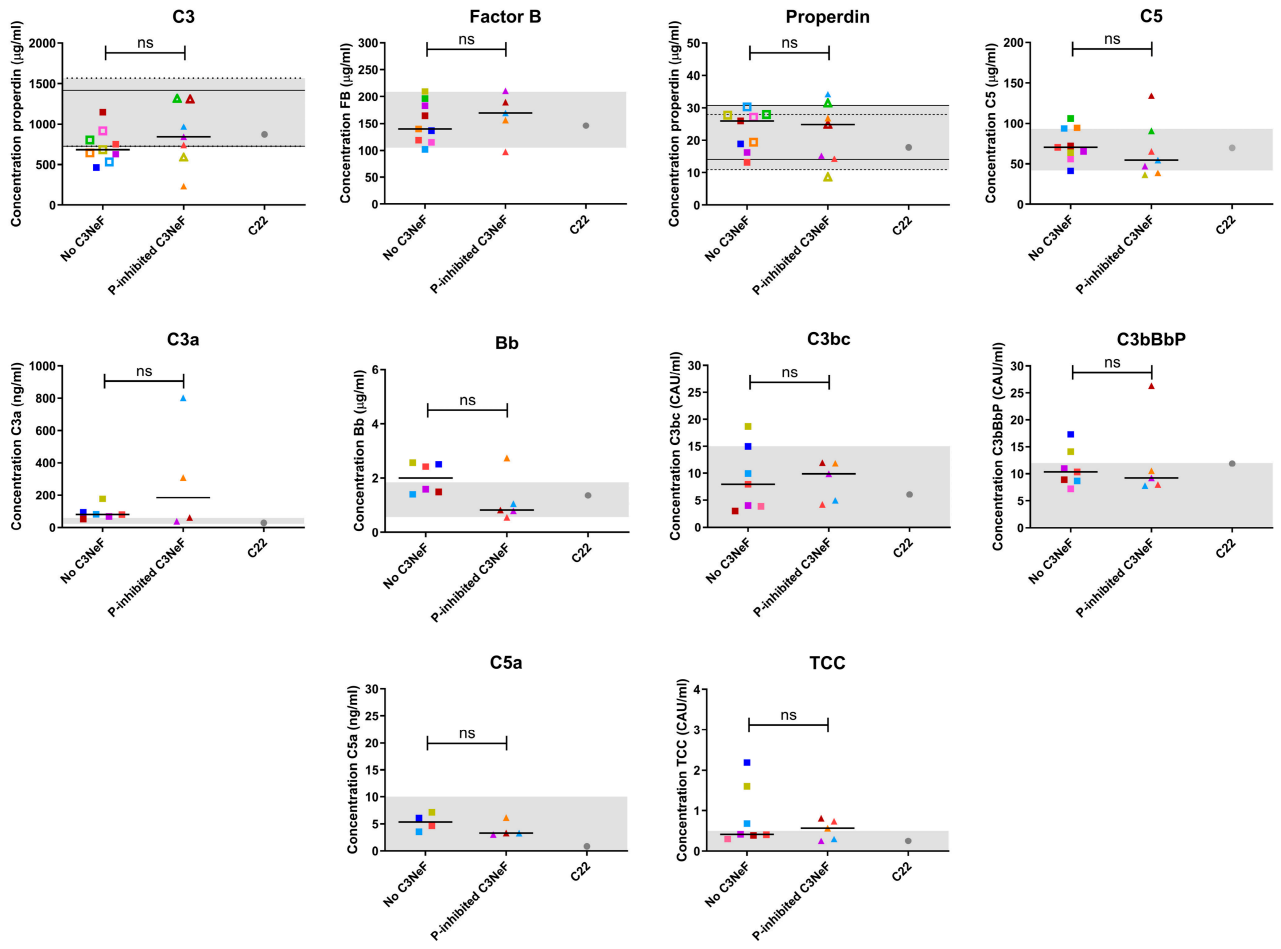
Concentrations of circulating complement proteins and activation products. Levels of the complement proteins C3, Factor B (FB), properdin, and C5 and activation products C3a, Bb, C3bc, C3bBbP, C5a, and terminal complement complex (TCC) were measured in samples of patients with C3G or IC-MPGN. The data points with the same symbol and color originate from the same patient. The median is given per group. For properdin and C3, data points measured in serum have open symbols and in EDTA-plasma have closed symbols. The reference values of the healthy controls are indicated by the grey areas. For properdin and C3, the dotted and continuous lines specify the reference values in serum and EDTA-plasma, respectively. Patients were categorized based on their outcome in the AP-CAA. C22 was positive for properdin-inhibited C3NeFs according to the AP-CAA but not included in the P-inhibited group for this analysis. Of note, the set of markers is not complete for all patients due to a lack of suitable material. Not significant (ns), using two-tailed Mann–Whitney U-tests. CAU, complement arbitrary.

To investigate potential functional effects of the properdin-inhibited C3NeFs, we examined the complement profiles of patients with properdin-inhibited C3NeFs, by measuring the complement components C3, FB, properdin, and C5, and complement activation products C3a, Bb, C3bc, C3bBbP, C5a, and TCC in the blood of patients. For this analysis, we used the classification into properdin-inhibited C3NeF and no C3NeF according to the results of the AP-CAA. No significant difference was observed between the C3NeF-negative and properdin-inhibited C3NeF group for all complement components and activation products (Figure 6). The properdin levels were largely within the normal range in all patients, including in the patients with properdin-inhibited C3NeFs. The healthy control with the properdin-inhibited C3NeF (C22) did not show aberrant complement activation or complement protein levels.

## 3. Discussion

C3NeFs are detected in more than half of patients with C3G and in a substantial proportion of IC-MPGN patients and are therefore considered drivers of complement dysregulation and disease pathogenesis. Improved characterization of C3G patients, including the C3NeFs they harbor, is essential for optimizing treatment strategies, particularly in the context of emerging complement-targeted therapies. Two classes of C3NeFs have already been well described: properdin-dependent and properdin-independent C3NeFs [17,34,38,39,40,41]. However, C3NeFs that stabilize the AP C3 convertase exclusively in the absence of properdin, referred to as properdin-inhibited C3NeFs, have remained largely unexplored. In this study, we examined whether properdin-inhibited C3NeFs are present in our C3G/IC-MPGN cohort, tried to further characterize them, and investigated their relation with complement markers in blood.

Using our rabbit erythrocyte-based AP-CAA, properdin-inhibited C3NeFs were detected in the sera of 7/16 patients (44%) from our C3G/IC-MPGN cohort consisting of patients who had previously tested negative for C3NeFs under physiological, properdin-containing serum conditions. These C3NeFs only became active in serum after properdin was blocked with Salp20. Thus, in our C3G/IC-MPGN cohort, around half of the patients who had previously been classified as negative for C3NeF activity in physiological serum conditions with properdin (described in ref. [46,47,48]) were now found to carry properdin-inhibited C3NeFs. This finding is consistent with the findings of Marinozzi et al. [17], who identified this type of C3NeF in 17/30 (57%) C3G patients who tested negative for NeFs in the presence of properdin. Even though a similar type of hemolytic sheep erythrocyte assay was used, Hauer et al. [42] detected properdin-inhibited C3NeFs in only 3/82 (4%) C3G patients who were negative in the C3bBbP(C3b) condition. Of note, properdin-inhibited C3NeFs are classified as single C3NeF positive samples in those studies, indicating no C5NeFs (term for NeFs recognizing the convertase with properdin, i.e., C3bBbP(C3b)) were found in those samples. Other studies that evaluated C3NeF activity under conditions both with and without properdin could not identify this specific properdin-inhibited C3NeF type [34,40,49]. In those studies, all samples positive for C3NeFs in the absence of properdin were also positive in the presence of properdin (properdin-independent C3NeFs). The high variation in prevalence across studies might be explained by differences in patient selection (e.g., C3G only versus C3G/IC-MPGN) and assay methodology (e.g., sheep erythrocyte- versus rabbit erythrocyte-based hemolytic assays or versus ELISA-based NeF assays). It is well-known in the field that C3NeF detection is highly assay-dependent, and that the same sample may yield different results depending on the assay used [34,40]. Therefore, comparing the prevalence of properdin-inhibited C3NeFs between cohort remains challenging.

The C3NeF activity in absence of properdin was confirmed with the purified Ig fractions in all five patients of whom material was available. However, the Ig fractions of three of the five patients also showed slightly prolonged convertase activity in the presence of properdin, i.e., when added to NHS. This is interesting because these patients were clearly negative for C3NeF activity when convertase activity was directly measured in the patient sera/plasmas. Nonetheless, these Igs showed relatively stronger convertase stabilization in the absence of properdin compared to the condition with properdin, so we still consider them as properdin-inhibited C3NeFs. For the three C3NeF-negative patients with Igs available, we confirmed that no convertase-stabilizing Igs were present, which is in line with the results found using the serum or plasma samples of these patients.

Findings obtained using the ELISA-based method demonstrate pronounced heterogeneity in properdin sensitivity among C3NeF-positive samples. The properdin-independent C3NeF-positive patients (P17, P18) showed high IC_50_ values indicating low sensitivity to properdin, suggesting that higher properdin concentrations are required to modulate convertase binding. In contrast, low IC_50_ samples were observed for the properdin-inhibited positive samples (P1, P12–14, and C22), indicating they are more responsive to properdin and lose their ability to bind to the C3 convertase upon the addition of properdin. These findings further support the presence of properdin-inhibited C3NeFs and likely reflect mechanistic differences in C3NeF–C3 convertase interactions and may have implications for stratification of patients. A possible explanation for decreased C3NeF binding might be that properdin-inhibited C3NeFs bind the AP C3 convertase at or near the region where properdin also binds [50,51,52], potentially creating competition between properdin and the C3NeF for binding the convertase. However, to date, this remains speculative and requires further experimental validation through crystal structures or more in-depth competition studies using, for example, ELISA or surface plasmon resonance. 

The AP-CAA with full serum containing properdin is used in our laboratory for routine patient testing. This assay did not detect the properdin-inhibited C3NeFs in the standard assay conditions. In contrast, the ELISA-based method did identify the presence of properdin-inhibited C3NeFs in the majority of the samples, both in the presence and absence of properdin. Despite the fact that decreased binding was observed when properdin was added to the properdin-inhibited C3NeF samples, five of the seven samples were still positive. Previous studies have demonstrated that the inclusion of properdin during convertase assessment enhances the number of positive samples [34,40,49], as it allows the detection of properdin-dependent C3NeF as well. In practice, however, the yield of positive C3NeFs may differ per assay. This further underlines that testing multiple test conditions provides most information on C3NeF presence and C3NeF type in study cohorts. Gaining insight into the different outcomes between methods, i.e., which assays can detect specific C3NeF types, and the underlying cause might improve our understanding of the low success rates of C3NeF detection assays [53] and improve patient characterization.

Surprisingly, we found that properdin-inhibited C3NeFs were also present in 1/23 healthy controls (C22; 4%), as determined by the AP-CAA. A previous study has reported the presence of C3NeFs in a healthy individual [54]. In this study [54], however, the C3NeFs were found in conditions with properdin, enabling the detection of properdin-dependent and properdin-independent C3NeFs but not properdin-inhibited C3NeFs. Generally, detecting C3NeFs in healthy persons raises questions about the clinical relevance of these C3NeFs. For the properdin-inhibited C3NeFs, this clinical and functional relevance may be questioned even more since in the presence of properdin—which reflect the physiological situation, at least in the circulation—these C3NeFs likely remain inactive and are unable to trigger complement-mediated disease, both in controls and in patients.

To gain further insight into the functional and physiological implications of properdin-inhibited C3NeFs, the complement activity profiles of patients carrying these autoantibodies were analyzed. We compared the complement profiles of the C3NeF-negative and properdin-inhibited C3NeF-positive patients and observed no significant differences between both groups. In addition, compared to our previous studies with properdin-(in)dependent C3NeFs [41,48], the complement profiles seemed to be less aberrant and less active in the properdin-inhibited C3NeF-positive patients. This generally normal complement profile may raise questions about whether properdin-inhibited C3NeFs are active in vivo, since the patients all had (near-)normal systemic properdin levels. This also applies to the healthy control with the properdin-inhibited C3NeFs (C22), who also showed a normal systemic properdin level and normal complement levels. However, it is also known that the correlations between C3NeF presence and complement activation profiles in blood are not perfect [21,48], so drawing conclusions about functional relevance from systemic complement levels alone may be misleading.

Furthermore, while our study assessed circulating properdin levels, it is important to emphasize that local properdin concentrations within the renal microenvironment might differ substantially from circulating levels, particularly during inflammation. For example, properdin can be locally produced by infiltrating leukocytes [55], which may prevent the stabilization of C3 convertases by properdin-inhibited C3NeFs. On the other hand, there are also conditions known in which properdin concentrations are decreased. For example, vaccination, infection, and cancer therapies are known the be related to decreased levels of leukocytes [56,57,58] and therefore lowered properdin levels. In these individuals, properdin-inhibited C3NeFs may become active, potentially contributing to the complement-mediated kidney damage in these patients, or they may even elicit such damage in healthy individuals carrying properdin-inhibited C3NeFs.

The characterization of the properdin-inhibited C3NeFs in C3G/IC-MPGN patients may become even more important in light of the upcoming complement-targeted therapeutics to treat complement-mediated kidney diseases. We previously showed that properdin-inhibiting therapy could be beneficial for those patients with properdin-dependent C3NeFs [41]. However, the same therapy could have dramatic pathogenic effects in patients with properdin-inhibited C3NeFs, in whom the C3NeF activity would be elicited. At the moment, no properdin inhibitors are in clinical trials for C3G/IC-MPGN, but properdin inhibition has been considered for other diseases in which the complement system plays a role [4,43,44,45]. If properdin-targeted therapies are considered for C3G/IC-MPGN in the future, particular caution will be warranted in patients harboring properdin-inhibited C3NeFs.

One of the primary limitations of this study was the relatively small sample size of both patients and controls. In total, 16 patients could be included, of whom 7 were classified as properdin-inhibited C3NeF positive, and experiments using purified Igs were available for only 5 of these 7 patients. Given these limited numbers, individual variability and potential outliers may have had a disproportionate influence on the observed results. In addition, detailed clinical data were unfortunately not available for all patients included in this retrospective study. Accordingly, the findings and clinical implications should be interpreted with caution. Further studies in larger, clinically well-described cohorts are needed to validate the prevalence and functional significance of properdin-inhibited C3NeFs. Another methodological limitation was the suboptimal curve fitting of the properdin titration in the anti-C3bBb(P) ELISA due to the limited number of data points. While the overall fits were acceptable, variability in goodness-of-fit and the limited number of concentration points indicate that curve fitting was not optimal for all samples. This may have affected the accuracy of the calculated IC_50_ and 95% confidence intervals. However, visual inspection of the data indicates a clear distinction between properdin-inhibited C3NeFs and properdin-independent C3NeFs, which supports the observed trend. Increasing the number and range of properdin concentrations would improve the accuracy and robustness of IC_50_ estimation in future experiments. A key strength of this study was the application of two distinct methodologies to investigate properdin-inhibited C3NeFs, enhancing the reliability of our findings.

In conclusion, we detected properdin-inhibited, Ig-based convertase-stabilizing factors, i.e., properdin-inhibited C3NeFs, in approximately 40% of the C3G/IC-MPGN patients who were negative for C3NeF activity in the presence of properdin. The vast majority of these properdin-inhibited C3NeF were also detected by an ELISA-based method detecting direct IgG binding to the C3 convertase. Upon addition of properdin, binding of properdin-inhibited C3NeFs decreased, suggesting that these C3NeFs indeed compete with or are affected by properdin. Despite the presence of these properdin-inhibited C3NeFs, we showed that the complement levels of these properdin-inhibited C3NeF-positive patients were not significantly different from C3NeF-negative patients. Especially since the systemic properdin levels in these individuals were generally normal, it could be questioned if the properdin-inhibited C3NeFs were active in these patients. However, in the light of complement inhibition, in particular properdin inhibition, the presence of these properdin-inhibited C3NeFs may lead to adverse effects. Further research on the functionality and clinical relevance of the properdin-inhibited C3NeFs is still required.

## 4. Materials and Methods

### 4.1. Sample Collection and Genetic Analysis

Serum and ethylenediamine-tetraacetic acid (EDTA)-plasma samples were collected from C3G and IC-MPGN patients referred to the Radboudumc and from healthy volunteers. Diagnosis was obtained from pathology reports following the guidelines described in the consensus report of the first C3G Meeting [14]. Subdivision of C3G into C3GN and DDD was based on electron microscopy appearance. Healthy controls were excluded in the case of fever, bacterial and/or viral infections in the preceding 2 weeks, chronic illness, inherited or acquired immune disorders, and immunosuppressive medication. The samples were processed as previously described [46]. The samples of the 23 healthy volunteers were used individually and were also pooled to obtain NHS and NHP. Heat-inactivated NHS (hi-NHS) was prepared by incubating NHS for 30 min at 56 °C.

Coding regions (including intron–exon boundaries) of complement (regulatory) genes were screened for rare genetic variants according to previously described protocol [48]. Genomic DNA, isolated from peripheral blood leukocytes, was amplified for *CFH*, *CFHR1*, *CFHR2*, *CFHR3*, *CFHR4*, *CFHR5*, *CFI*, *CD46*, *C3*, and *CFB* by means of PCR, followed by sequence analysis and screening for rare genetic variants (minor allele frequency < 1%). Only genetic variants of unknown significance, likely pathogenic, or pathogenic (according to guidelines of the American College of Medical and Genomics [59]) are mentioned. Multiplex ligation-dependent probe amplification (MLPA; 42 probes) was used to screen for rearrangements in the *CFH/CFHR* region. MLPA was not analyzed for P1, P2, P5, and P6. *CFHR1-5* genes were not analyzed for P2, P5–7, and P9. No genetic analysis was performed for P4 and P11.

### 4.2. Immunoglobulin Purification

The Ig fractions from seven patients, nine healthy controls, and two control pools were purified following previously described protocol [46], using Nab™ protein A/G spin columns (Thermo Fisher Scientific, Waltham, MA, USA). Igs were isolated from EDTA-plasma (P1, P2, P8, P9, P13, P14, P16, C1, C4, C5, C12, C15, C18, C20, C22, C23, and NHP) or serum (P12 and NHS) samples, based on availability. After purification and dialysis, Ig fractions were concentrated in phosphate-buffered saline to the initial sample volume.

### 4.3. Alternative Pathway Convertase Activity Assay

The AP-CAA, which has been described previously [46], is a functional hemolytic assay for measuring C3NeF activity. The hemolytic assay is separated into two steps by the C5-blocking agent eculizumab. In the first step, AP convertases are assembled. In the second step, the activity of these convertases is quantified using C5b-9-mediated hemolysis. The AP convertase activity is monitored at different time points, making visualization of convertase stability possible.

For this study, we used two previously described approaches for the detection of convertase-stabilizing factors in conditions with and without properdin [41]. In the first method, we tested convertase stability in patient serum or EDTA-plasma treated with or without the properdin inhibitor Salp20. Patient samples were always mixed with an equal volume of NHS to compensate for possible low C3 levels [41]. P3 and P13 required extra addition of 500 µg/mL C3 (A113; Complement Technology, Tyler, TX, USA) to obtain interpretable convertase activity profiles [41]. Where indicated, Salp20 was added to the test sample in a final concentration of 6.25 µg/mL per volume of undiluted serum. Salp20 was obtained according to a previously described protocol [41]. In the second method, we examined the convertase-stabilizing ability of Igs purified from patient samples when added to NHS or ΔP-NHS. ΔP-NHS was obtained from Complement Technology (A339). NHS and ΔP-NHS were supplemented with Ig fractions from controls or patients in a 1:3 volume ratio.

To correct for the experimental variation from the used rabbit erythrocyte batches in this study, we compared the hemolysis levels of the test samples to the hemolysis obtained by the internal control taken along in each experiment, i.e., NHS or NHS + Salp20, or NHS or ΔP-NHS supplemented with NHP or NHS Igs. The deviation of the hemolysis of the test sample to the hemolysis of the internal control is referred to as the relative deviation.

To define cut-off values, the hemolysis levels were assessed at the time points at which controls showed background hemolysis, i.e., t30 and t40 for conditions with properdin and t50 and t60 for conditions without properdin. A sample was considered positive for prolonged convertase activity when it met the cut-off criteria for each test condition below, i.e., when the relative deviation of the test sample exceeded the mean relative deviation + 3SD of the hemolysis obtained by the healthy controls (Figure 2A,B and Figure S1A,B). Control samples outside Q1 − 1.5 IQR or Q3  +  1.5 IQR for the condition with serum and Salp20 were considered as outliers and excluded for the calculation of all cut-off values.

$$\begin{array}{c} \mathrm{Complete}\;\mathrm{serum}:\;\frac{Average\;hemolysis\;t30+40\;of\;patient}{Average\;hemolysis\;t30+40\;of\;NHS}>\text{1.68 (mean relative deviation + 3SD of healthy controls)}\\ \text{Mean 1.07, SD 0.20} \end{array}$$

$$\begin{array}{c} \mathrm{Complete}\;\mathrm{serum}\;\mathrm{with}\;\mathrm{Salp}20:\;\frac{Average\;hemolysis\;t50+60\;of\;patient+Salp20}{Average\;hemolysis\;t50+60\;of\;NHS+Salp20}>\text{1.20 (mean relative deviation + 3SD of healthy controls)}\\ \text{Mean 0.66, SD 0.18} \end{array}$$

$$\begin{array}{c} \mathrm{NHS}\;\mathrm{with}\;\mathrm{Ig}\;\mathrm{fractions}:\;\frac{Average\;hemolysis\;t30+40\;of\;NHS+patient\;Igs}{Average\;hemolysis\;t30+40\;of\;NHS+NHS/NHP\;Igs}>\text{1.36 (mean relative deviation + 3SD of healthy controls)}\\ \text{Mean 1.00, SD 0.12} \end{array}$$

$$\begin{array}{c} \Delta P\text{-}\mathrm{NHS}\;\mathrm{with}\;\mathrm{Ig}\;\mathrm{fractions}:\;\frac{Average\;hemolysis\;t50+60\;of\;\Delta P\text{-}NHS+patient\;Igs}{Average\;hemolysis\;t50+60\;of\;\Delta P\text{-}NHS+NHS/NHP\;Igs}>\text{1.95 (mean relative deviation + 3SD of healthy controls)}\\ \text{Mean 1.16, SD 0.26} \end{array}$$

### 4.4. Anti-C3bBb(P) Detection

IgG binding to the C3 convertase was detected using a newly developed ELISA (cat. #HK3051; Hycult Biotech, Uden, The Netherlands). The assay was performed according to the manufacturer’s instructions. In short, C3b-precoated microtiter wells were incubated with hi-serum or hi-EDTA plasma (0.5% per well) diluted in assay-specific dilution buffer, together with FB and FD to allow C3 convertase formation (C3bBb) and binding by C3NeFs. Where indicated, 0.5–4 µg/mL properdin (A139, Complement Technology) was added together with FB and FD to assess C3bBbP binding. Samples were incubated for 30 min at 37 °C. After washing, HRP-conjugated goat anti-human IgG antibody in dilution buffer was added and plates were incubated for 30 min at RT. Plates were washed (4×) and TMB substrate was added. The peroxidase reaction was stopped after 15 min, using 2% (*w*/*v*) oxalic acid. Absorbance was measured at 450 nm using SPARK^®^ multimode microplate reader (Tecan, Giessen, The Netherlands). To identify convertase-specific binding, delta absorbance values (ΔA_405_) were calculated by subtracting the absorbance of the assay blank of the test sample (A_405_C3b) from the absorbance of the test well of this sample (A_405_C3bBb and A_405_C3bBbP). The cut-off for positive convertase binding was determined by the mean + 3 SD of the ΔA_405_ from 22 healthy individuals.

### 4.5. Quantification of Complement Proteins and Activation Products

Levels of the activation markers C3bBbP, C3bc, and TCC [41,60,61] and complement proteins C5 [41] and properdin [47] were measured in the appropriate EDTA-plasma or serum samples with ELISAs as previously described. FB levels were measured in human serum or EDTA-plasma using a polyclonal goat anti-human FB antibody (ABIN594942; antibodies-online, Aachen, Germany), combined with a horseradish peroxidase-conjugated monoclonal mouse anti-human FB antibody (ABIN933687; antibodies-online). The plate was developed with TMB (T2885; Sigma-Aldrich, St. Louis, MO, USA) and results were calculated based on a calibration line produced by the commercially obtained purified human FB standard (A135; Complement Technology). C3 levels were measured in human serum or EDTA-plasma using polyclonal antiserum against C3 (A213, Complement Technology), combined with a horseradish peroxidase-conjugated goat anti-C3 antibody (55237; MP Biomedicals, Santa Ana, CA, USA). The plate was developed with OPD and results were calculated based on a calibration line produced by the commercially obtained purified human C3b standard (204860; Merck Millipore, Darmstadt, Germany). Bb, C3a, and C5a levels were measured in EDTA-plasma using commercial kits: MicroVue Bb Plus EIA (Quidel, San Diego, CA, USA), MicroVue Complement C3a Plus EIA (Quidel, San Diego, CA, USA), and Human Complement Component C5a DuoSet ELISA (R&D systems, Minneapolis, MN, USA), respectively.

### 4.6. Statistics and Data Analysis

For statistical analysis and data visualization, GraphPad Prism 10.6.1 for Windows was used (GraphPad Software, San Diego, CA, USA). For the analysis of the ELISA-based dose–response assay with increasing concentrations of properdin, normalized data compared to 0 µg/mL properdin was used. Data were log-transformed and fitted using nonlinear regression (three-parameter logistic model). IC_50_ values and corresponding 95% confidence intervals were derived from the fitted curves. Differences in properdin sensitivity between samples were evaluated using an extra sum-of-squares F test comparing models with shared versus individual LogIC_50_ values. For the other analyses, data were analyzed using Kruskal–Wallis test (Dunn’s multiple comparisons test) or two-tailed Mann–Whitney U-tests, where indicated. Differences achieving *p* < 0.05 were considered statistically significant.

## Figures and Tables

**Table 1:** Patient characteristics. All genetic variants found were heterozygous. C3GN, C3 glomerulonephritis; DDD, dense deposit disease; IC-MPGN, immune complex-mediated membranoproliferative glomerulonephritis; NA, not available; N, no; Y, yes.

Patient	Sex (M/F)	Age at Time of First Presentation (Years)	Age at Time of Study (Years)	C3G Subtype	Genetic Aberrations	Autoantibodies Against Factor H (Y/N)
C3G						
P1	F	17	17	DDD	-	N
P2	F	22	22	DDD	-	N
P3	M	9	10	NA	-	N
P4	F	7	9	DDD	NA	N
P5	M	42	42	DDD	C3 c.26T > C (p.Leu9Pro)	N
P6	M	62	65	DDD	-	N
P7	F	33	33	NA	-	N
P8	M	66	66	C3GN	-	N
P9	M	43	44	C3GN	-	N
P10	M	29	37	C3GN	-	N
P11	F	42	42	C3GN	NA	NA
P12	F	5	12	DDD	-	N
IC-MPGN						
P13	F	14	14	-	-	N
P14	F	17	17	-	-	N
P15	M	18	19	-	-	N
P16	F	15	16	-	-	N

**Table 2:** Half maximal inhibitory concentration (IC_50_) values and 95% confidence intervals obtained from nonlinear regression of properdin titration in ELISA for convertase binding. For each sample, data were derived from three independent measurements.

	C3NeF Classification According to AP-CAA	IC_50_ (µg/mL)	95% Confidence Interval (µg/mL)
C22	Properdin-inhibited	0.98	0.69–1.36
P1	Properdin-inhibited	1.31	1.03–1.68
P12	Properdin-inhibited	1.01	0.72–1.40
P13	Properdin-inhibited	1.35	1.07–1.69
P14	Properdin-inhibited	3.69	2.60–5.35
P17	Properdin-independent	10.17	8.84–11.84
P18	Properdin-independent	8.63	6.15–12.91

## Data Availability

The original contributions presented in this study are included in the article/Supplementary Materials. Further inquiries can be directed to the corresponding author.

## Supplementary Materials

The following supporting information can be downloaded at: https://www.mdpi.com/article/10.3390/ijms27156790/s1.

## References

[1] Ricklin D., Hajishengallis G., Yang K., Lambris J.D. (2010). Complement: A key system for immune surveillance and homeostasis. Nat. Immunol..

[2] Merle N.S., Church S.E., Fremeaux-Bacchi V., Roumenina L.T. (2015). Complement system part I—Molecular mechanisms of activation and regulation. Front. Immunol..

[3] Lesher A.M., Nilsson B., Song W.C. (2013). Properdin in complement activation and tissue injury. Mol. Immunol..

[4] Michels M., Volokhina E.B., van de Kar N., van den Heuvel L. (2019). The role of properdin in complement-mediated renal diseases: A new player in complement-inhibiting therapy?. Pediatr. Nephrol..

[5] Fearon D.T., Austen K.F. (1975). Properdin: Binding to C3b and stabilization of the C3b-dependent C3 convertase. J. Exp. Med..

[6] Berends E.T., Gorham R.D., Ruyken M., Soppe J.A., Orhan H., Aerts P.C., de Haas C.J., Gros P., Rooijakkers S.H. (2015). Molecular insights into the surface-specific arrangement of complement C5 convertase enzymes. BMC Biol..

[7] Michels M., Maas R.J.F., van der Velden T., van de Kar N., van den Heuvel L., Volokhina E.B. (2021). The role of properdin in C5 convertase activity and C5b-9 formation in the complement alternative pathway. J. Immunol..

[8] Zipfel P.F., Skerka C. (2009). Complement regulators and inhibitory proteins. Nat. Rev. Immunol..

[9] Ricklin D., Reis E.S., Lambris J.D. (2016). Complement in disease: A defence system turning offensive. Nat. Rev. Nephrol..

[10] Noris M., Remuzzi G. (2013). Overview of complement activation and regulation. Semin. Nephrol..

[11] Smith R.J.H., Appel G.B., Blom A.M., Cook H.T., D’Agati V.D., Fakhouri F., Fremeaux-Bacchi V., Józsi M., Kavanagh D., Lambris J.D. (2019). C3 glomerulopathy—Understanding a rare complement-driven renal disease. Nat. Rev. Nephrol..

[12] Goodship T.H., Cook H.T., Fakhouri F., Fervenza F.C., Frémeaux-Bacchi V., Kavanagh D., Nester C.M., Noris M., Pickering M.C., Rodríguez de Córdoba S. (2017). Atypical hemolytic uremic syndrome and C3 glomerulopathy: Conclusions from a “Kidney Disease: Improving Global Outcomes” (KDIGO) Controversies Conference. Kidney Int..

[13] Zipfel P.F., Skerka C., Chen Q., Wiech T., Goodship T., Johnson S., Fremeaux-Bacchi V., Nester C., de Córdoba S.R., Noris M. (2015). The role of complement in C3 glomerulopathy. Mol. Immunol..

[14] Pickering M.C., D’Agati V.D., Nester C.M., Smith R.J., Haas M., Appel G.B., Alpers C.E., Bajema I.M., Bedrosian C., Braun M. (2013). C3 glomerulopathy: Consensus report. Kidney Int..

[15] Hou J., Markowitz G.S., Bomback A.S., Appel G.B., Herlitz L.C., Barry Stokes M., D’Agati V.D. (2014). Toward a working definition of C3 glomerulopathy by immunofluorescence. Kidney Int..

[16] Medjeral-Thomas N.R., O’Shaughnessy M.M., O’Regan J.A., Traynor C., Flanagan M., Wong L., Teoh C.W., Awan A., Waldron M., Cairns T. (2014). C3 glomerulopathy: Clinicopathologic features and predictors of outcome. Clin. J. Am. Soc. Nephrol..

[17] Marinozzi M.C., Chauvet S., Le Quintrec M., Mignotet M., Petitprez F., Legendre C., Cailliez M., Deschenes G., Fischbach M., Karras A. (2017). C5 nephritic factors drive the biological phenotype of C3 glomerulopathies. Kidney Int..

[18] Iatropoulos P., Daina E., Curreri M., Piras R., Valoti E., Mele C., Bresin E., Gamba S., Alberti M., Breno M. (2018). Cluster analysis identifies distinct pathogenetic patterns in C3 glomerulopathies/immune complex-mediated membranoproliferative GN. J. Am. Soc. Nephrol..

[19] Rabasco C., Cavero T., Román E., Rojas-Rivera J., Olea T., Espinosa M., Cabello V., Fernández-Juarez G., González F., Ávila A. (2015). Effectiveness of mycophenolate mofetil in C3 glomerulonephritis. Kidney Int..

[20] Lu D.F., Moon M., Lanning L.D., McCarthy A.M., Smith R.J. (2012). Clinical features and outcomes of 98 children and adults with dense deposit disease. Pediatr. Nephrol..

[21] Servais A., Noël L.H., Roumenina L.T., Le Quintrec M., Ngo S., Dragon-Durey M.A., Macher M.A., Zuber J., Karras A., Provot F. (2012). Acquired and genetic complement abnormalities play a critical role in dense deposit disease and other C3 glomerulopathies. Kidney Int..

[22] Bomback A.S., Santoriello D., Avasare R.S., Regunathan-Shenk R., Canetta P.A., Ahn W., Radhakrishnan J., Marasa M., Rosenstiel P.E., Herlitz L.C. (2018). C3 glomerulonephritis and dense deposit disease share a similar disease course in a large United States cohort of patients with C3 glomerulopathy. Kidney Int..

[23] Riedl M., Thorner P., Licht C. (2017). C3 Glomerulopathy. Pediatr. Nephrol..

[24] Attieh R.M., Bharati J., Sharma P., Nair G., Ayehu G., Jhaveri K.D. (2025). Kidney transplant in patients with C3 glomerulopathy. Clin. Kidney J..

[25] (2021). Kidney Disease: Improving Global Outcomes (KDIGO) Glomerular Diseases Work Group. KDIGO 2021 Clinical Practice Guideline for the Management of Glomerular Diseases. Kidney Int..

[26] Tarragon Estebanez B., Bomback A.S. (2024). C3 glomerulopathy: Novel treatment paradigms. Kidney Int. Rep..

[27] Caliskan Y., Torun E.S., Tiryaki T.O., Oruc A., Ozluk Y., Akgul S.U., Temurhan S., Oztop N., Kilicaslan I., Sever M.S. (2017). Immunosuppressive treatment in C3 glomerulopathy: Is it really effective?. Am. J. Nephrol..

[28] Kavanagh D., Ariceta G., Vivarelli M., Schaefer F., Caravaca-Fontán F., Frémeaux-Bacchi V., Fakhouri F., Licht C., Pickering M.C. (2026). Current and emerging therapies for C3 glomerulopathy and primary (idiopathic) immune complex membranoproliferative glomerulonephritis. Kidney Int. Rep..

[29] Mashayekhi M., Zuckerman J.E., Barratt J., Glassock R.J., Caravaca-Fontán F., Hsu R.K., Rajasekaran A., Lerma E., Noor S.M., Abdipour A. (2026). C3 glomerulopathy diagnosis, current Treatments, and emerging therapies. Kidney Med..

[30] Meuleman M.S., Vieira-Martins P., El Sissy C., Audard V., Baudouin V., Bertrand D., Bridoux F., Louillet F., Dossier C., Esnault V. (2023). Rare variants in complement gene in C3 glomerulopathy and immunoglobulin-mediated membranoproliferative GN. Clin. J. Am. Soc. Nephrol..

[31] Michels M., Volokhina E.B., van de Kar N., van den Heuvel L. (2022). Challenges in diagnostic testing of nephritic factors. Front. Immunol..

[32] Ito S., Tamura N., Fujita T. (1989). Effect of decay-accelerating factor on the assembly of the classical and alternative pathway C3 convertases in the presence of C4 or C3 nephritic factor. Immunology.

[33] Ohi H., Watanabe S., Fujita T., Yasugi T. (1992). Significance of C3 nephritic factor (C3NeF) in non-hypocomplementaemic serum with membranoproliferative glomerulonephritis (MPGN). Clin. Exp. Immunol..

[34] Paixão-Cavalcante D., López-Trascasa M., Skattum L., Giclas P.C., Goodship T.H., de Córdoba S.R., Truedsson L., Morgan B.P., Harris C.L. (2012). Sensitive and specific assays for C3 nephritic factors clarify mechanisms underlying complement dysregulation. Kidney Int..

[35] Daha M.R., Kok D.J., Van Es L.A. (1982). Regulation of the C3 nephritic factor stabilized C3/C5 convertase of complement by purified human erythrocyte C3b receptor. Clin. Exp. Immunol..

[36] Noris M., Donadelli R., Remuzzi G. (2019). Autoimmune abnormalities of the alternative complement pathway in membranoproliferative glomerulonephritis and C3 glomerulopathy. Pediatr. Nephrol..

[37] Chae D.W. (2014). New classification of membranoproliferative glomerulonephritis: A good start but a long way to go. Kidney Res. Clin. Pract..

[38] Tanuma Y., Ohi H., Hatano M. (1990). Two types of C3 nephritic factor: Properdin-dependent C3NeF and properdin-independent C3NeF. Clin. Immunol. Immunopathol..

[39] Clardy C.W., Forristal J., Strife C.F., West C.D. (1989). A properdin dependent nephritic factor slowly activating C3, C5, and C9 in membranoproliferative glomerulonephritis, types I and III. Clin. Immunol. Immunopathol..

[40] Zhang Y., Meyer N.C., Wang K., Nishimura C., Frees K., Jones M., Katz L.M., Sethi S., Smith R.J. (2012). Causes of alternative pathway dysregulation in dense deposit disease. Clin. J. Am. Soc. Nephrol..

[41] Michels M., van de Kar N., van den Bos R.M., van der Velden T., van Kraaij S.A.W., Sarlea S.A., Gracchi V., Oosterveld M.J.S., Volokhina E.B., van den Heuvel L. (2019). Novel assays to distinguish between properdin-dependent and properdin-independent C3 nephritic factors provide insight into properdin-inhibiting therapy. Front. Immunol..

[42] Hauer J.J., Zhang Y., Goodfellow R., Taylor A., Meyer N.C., Roberts S., Shao D., Fergus L., Borsa N.G., Hall M. (2024). Defining nephritic factors as diverse drivers of systemic complement dysregulation in C3 Glomerulopathy. Kidney Int. Rep..

[43] Dai Y., Hill K., Harris D., Shen T., Yuan C.-X., Gasteyger C. (2022). A phase 2a, randomized, open-label study to evaluate multiple dosing regimens of subcutaneous ALXN1820 in adult patients with sickle cell disease. Blood.

[44] Glazer L.C., Williams J.G., Gordon C.M., Dugel P.U., Milton M., Valencia T., Klein U., Kretz S., Gedif K., Grosskreutz C.L. (2016). A first in human study of Intravitreal (IVT) CLG561 in subjects with Advanced Age-Related Macular Degeneration (AMD). Investig. Ophthalmol. Vis. Sci..

[45] Sandhu A., Shen T., Herrero P.M., Yuan C.X., Qureshi S., Jiang X., Sheng Y., Gasteyger C., Dai Y. (2025). Safety, tolerability, pharmacokinetics, pharmacodynamics, and immunogenicity of ALXN1820 (tarperprumig) in healthy adults: Results of a phase I study. Clin. Transl. Sci..

[46] Michels M., van de Kar N., Okrój M., Blom A.M., van Kraaij S.A.W., Volokhina E.B., van den Heuvel L. (2018). Overactivity of alternative pathway convertases in patients with complement-mediated renal diseases. Front. Immunol..

[47] Michels M., van de Kar N., van Kraaij S.A.W., Sarlea S.A., Gracchi V., Engels F., Dorresteijn E.M., van der Deure J., Duineveld C., Wetzels J.F.M. (2021). Different aspects of classical pathway overactivation in patients with C3 glomerulopathy and immune complex-mediated membranoproliferative glomerulonephritis. Front. Immunol..

[48] Michels M., Wijnsma K.L., Kurvers R.A.J., Westra D., Schreuder M.F., van Wijk J.A.E., Bouts A.H.M., Gracchi V., Engels F., Keijzer-Veen M.G. (2022). Long-term follow-up including extensive complement analysis of a pediatric C3 glomerulopathy cohort. Pediatr. Nephrol..

[49] Donadelli R., Pulieri P., Piras R., Iatropoulos P., Valoti E., Benigni A., Remuzzi G., Noris M. (2018). Unraveling the molecular mechanisms underlying complement dysregulation by nephritic factors in C3G and IC-MPGN. Front. Immunol..

[50] Pedersen D.V., Gadeberg T.A.F., Thomas C., Wang Y., Joram N., Jensen R.K., Mazarakis S.M.M., Revel M., El Sissy C., Petersen S.V. (2019). Structural basis for properdin oligomerization and convertase stimulation in the human complement system. Front. Immunol..

[51] Alcorlo M., Tortajada A., Rodríguez de Córdoba S., Llorca O. (2013). Structural basis for the stabilization of the complement alternative pathway C3 convertase by properdin. Proc. Natl. Acad. Sci. USA.

[52] van den Bos R.M., Pearce N.M., Granneman J., Brondijk T.H.C., Gros P. (2019). Insights Into enhanced complement activation by structures of properdin and its complex with the C-terminal domain of C3b. Front. Immunol..

[53] Kirschfink M., Frazer-Abel A., Balogh E., Goseberg S., Weiss N., Prohászka Z. (2024). External quality assurance program for diagnostic complement laboratories: Evaluation of the results of the past seven years. Front. Immunol..

[54] Egan M., Sullivan K., Frazer-Abel A., Cunningham-Rundles C. (2016). A healthy female with C3 hypocomplementemia and C3 Nephritic Factor. Clin. Immunol..

[55] Cortes C., Ohtola J.A., Saggu G., Ferreira V.P. (2012). Local release of properdin in the cellular microenvironment: Role in pattern recognition and amplification of the alternative pathway of complement. Front. Immunol..

[56] Frater J.L. (2020). How I investigate neutropenia. Int. J. Lab. Hematol..

[57] Palmblad J., Nilsson C.C., Höglund P., Papadaki H.A. (2016). How we diagnose and treat neutropenia in adults. Expert Rev. Hematol..

[58] Muturi-Kioi V., Lewis D., Launay O., Leroux-Roels G., Anemona A., Loulergue P., Bodinham C.L., Aerssens A., Groth N., Saul A. (2016). Neutropenia as an adverse event following vaccination: Results from randomized clinical trials in healthy adults and systematic review. PLoS ONE.

[59] Richards S., Aziz N., Bale S., Bick D., Das S., Gastier-Foster J., Grody W.W., Hegde M., Lyon E., Spector E. (2015). Standards and guidelines for the interpretation of sequence variants: A joint consensus recommendation of the American College of Medical Genetics and Genomics and the Association for Molecular Pathology. Genet. Med..

[60] Volokhina E.B., Westra D., van der Velden T.J., van de Kar N.C., Mollnes T.E., van den Heuvel L.P. (2015). Complement activation patterns in atypical haemolytic uraemic syndrome during acute phase and in remission. Clin. Exp. Immunol..

[61] Bergseth G., Ludviksen J.K., Kirschfink M., Giclas P.C., Nilsson B., Mollnes T.E. (2013). An international serum standard for application in assays to detect human complement activation products. Mol. Immunol..

