# Supplementary material for: Characterizing Properdin-Inhibited C3 Nephritic Factors in Patients with Complement-Mediated Kidney Diseases
Source: Int J Mol Sci. 2026 Jul 29;27(15):6790. doi: 10.3390/ijms27156790 (PMC13466216; doi:10.3390/ijms27156790)
Supplement: Supplementary file 1 [file ijms-27-06790-s001.zip › ijms-4322097-supplementary.pdf]

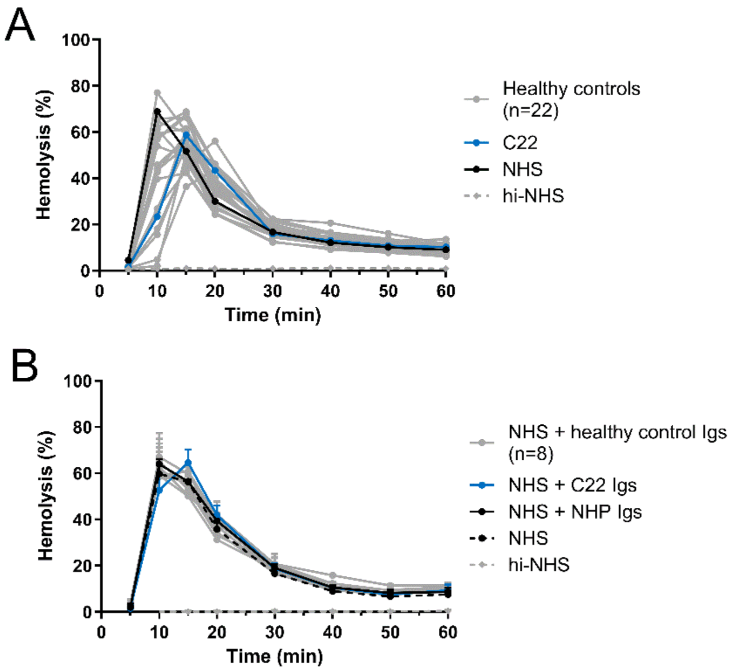

Suppl. Figure S1. Convertase activity profiles of healthy controls in the presence of properdin. The alternative pathway (AP)-convertase activity assay (AP-CAA) was performed with healthy control serum **(A)** or with immunoglobulins (Igs) fractions added to normal human serum (NHS) **(B)**. As an internal control, Igs originating from normal human plasma (NHP) were used. The healthy controls used for determination of cut-off values are shown in grey. Data are presented as a single experiment **(A)** or the mean + range of two independent experiments **(B)**. hi-NHS, heat-inactivated NHS.

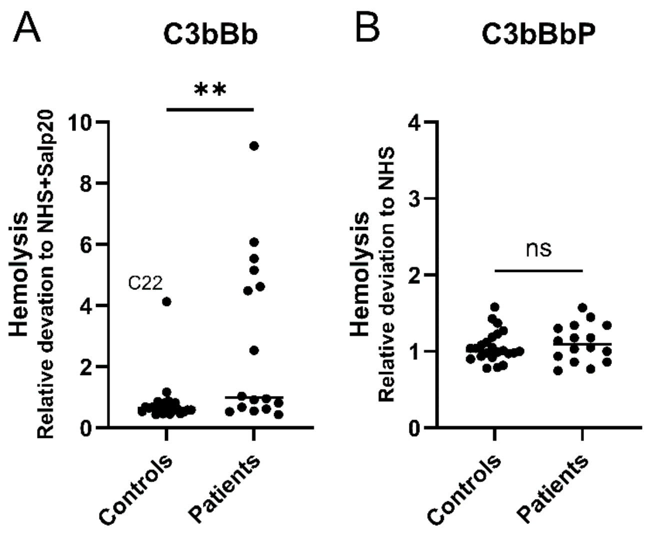

Suppl. Figure S2. Convertase activity of controls and patient measured with the AP-CAA. (A) Relative deviation to NHS+Salp20 (average t50+t60) in the absence of properdin. **(B)** Relative deviation to NHS (average t30+t40) in the presence of properdin. The median is given per group. Not significant (ns), **P < 0.01, using Mann-Whitney U-test.
